# Supplementary material for: A framework to predict the price of energy for the end-users with applications to monetary and energy policies
Source: Nat Commun. 2021 Jan 4;12:18. doi: 10.1038/s41467-020-20203-2 (PMC7782726; doi:10.1038/s41467-020-20203-2)
Supplement: Supplementary file 1 — Supplementary Information [file 41467_2020_20203_MOESM1_ESM.pdf]

Supporting information material: A framework to predict the price of energy for the end-users with applications to monetary and energy policies

Stefanos G. Baratsas[1,2], Alexander M. Niziolek[1,2], Onur Onel[1,2], Logan R. Matthews[1,2], Christodoulos A. Floudas[1,2], Detlef R. Hallermann[3], Sorin M. Sorescu[3], and Efstratios N. Pistikopoulos[1,2,*]

[1]Artie McFerrin Department of Chemical Engineering, Texas A&M University, College Station, TX 77843, United States
[2]Texas A&M Energy Institute, Texas A&M University, College Station, TX 77843, United States
[3]Department of Finance, Mays Business School, Texas A&M University, College Station, TX 77843, United States
[*]Corresponding author: stratos@tamu.edu

Supplementary Information

Supplementary Figures

Supplementary Figure 1a: Landscape of Crude Oil in the United States

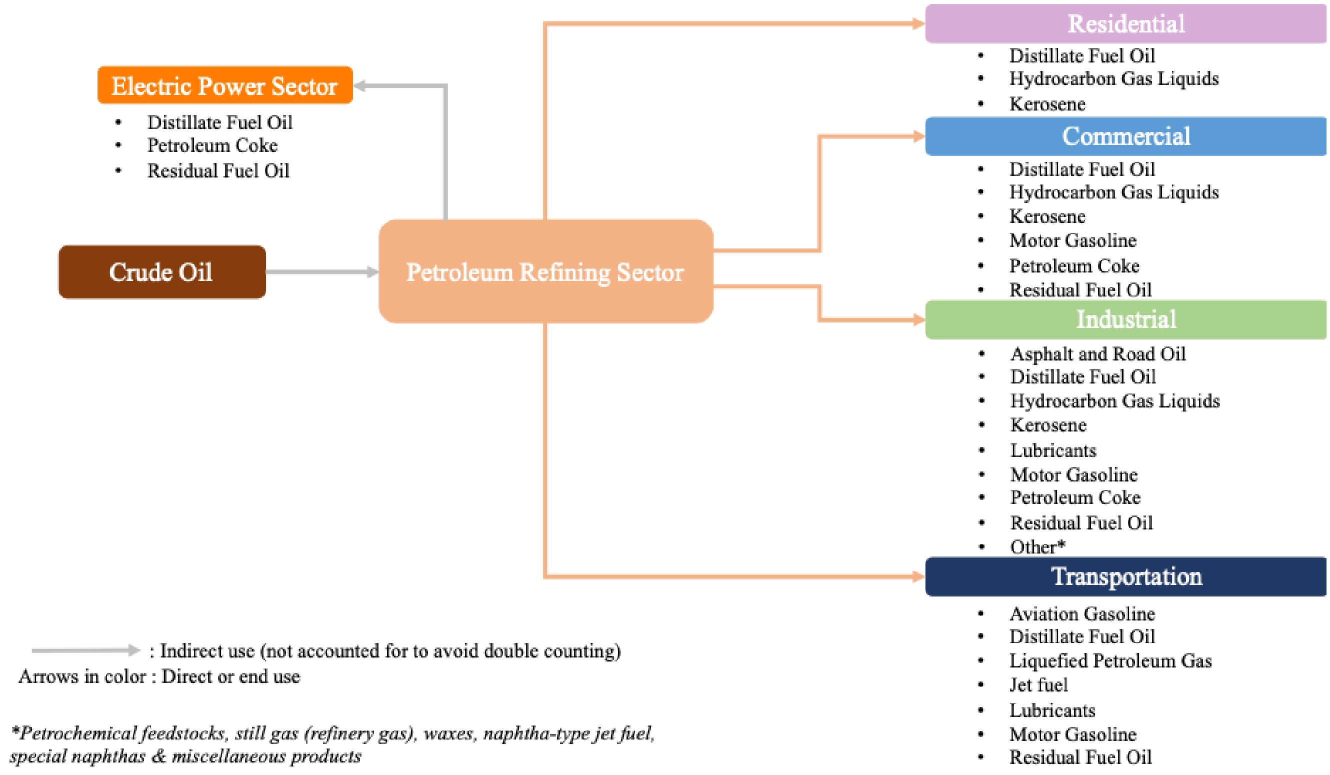

Supplementary Figure 1b: Landscape of Natural Gas and Coal in the United States

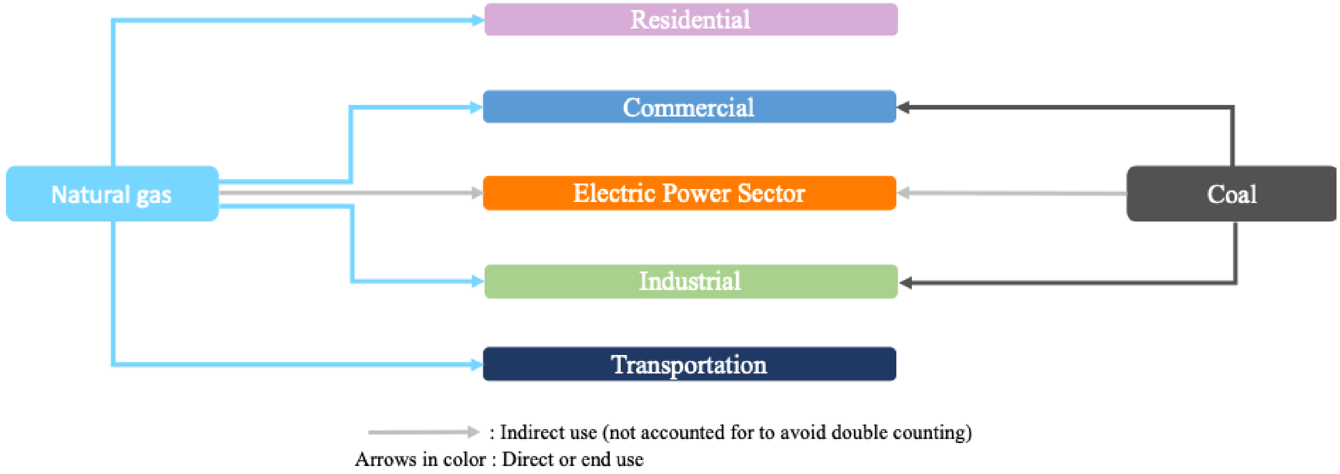

17 **Supplementary Figure 1c: Landscape of Solar and Wind in the United States**

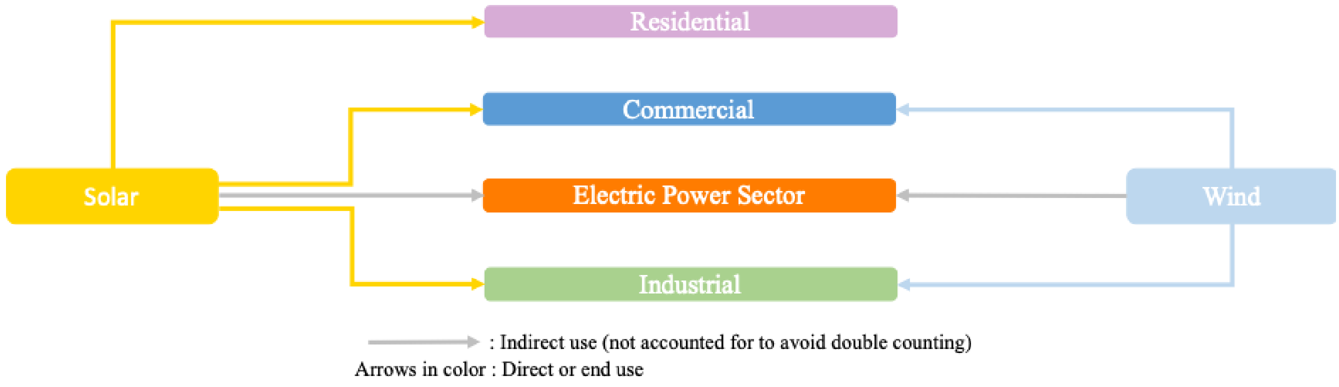

18 **Supplementary Figure 1d: Landscape of Geothermal and Hydroelectric in the United States**

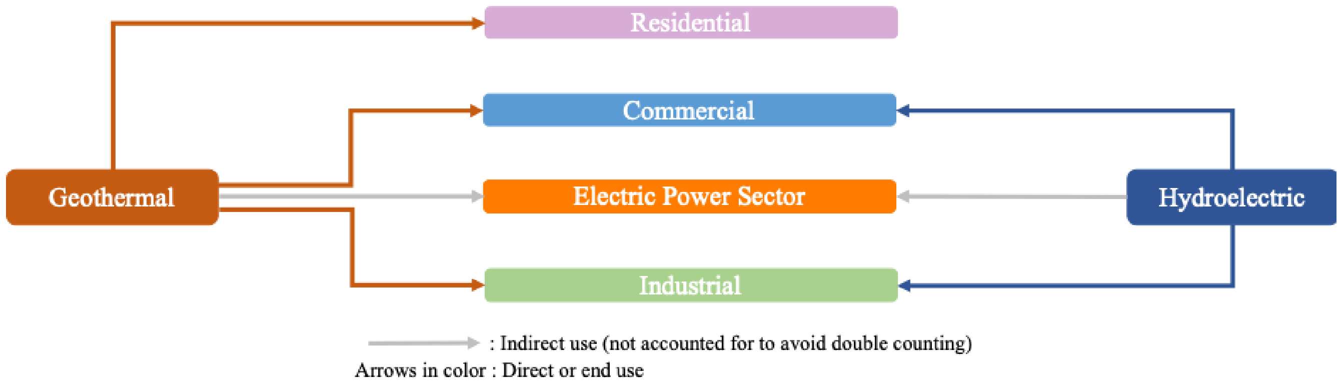

19 **Supplementary Figure 1e: Landscape of Biomass in the United States**

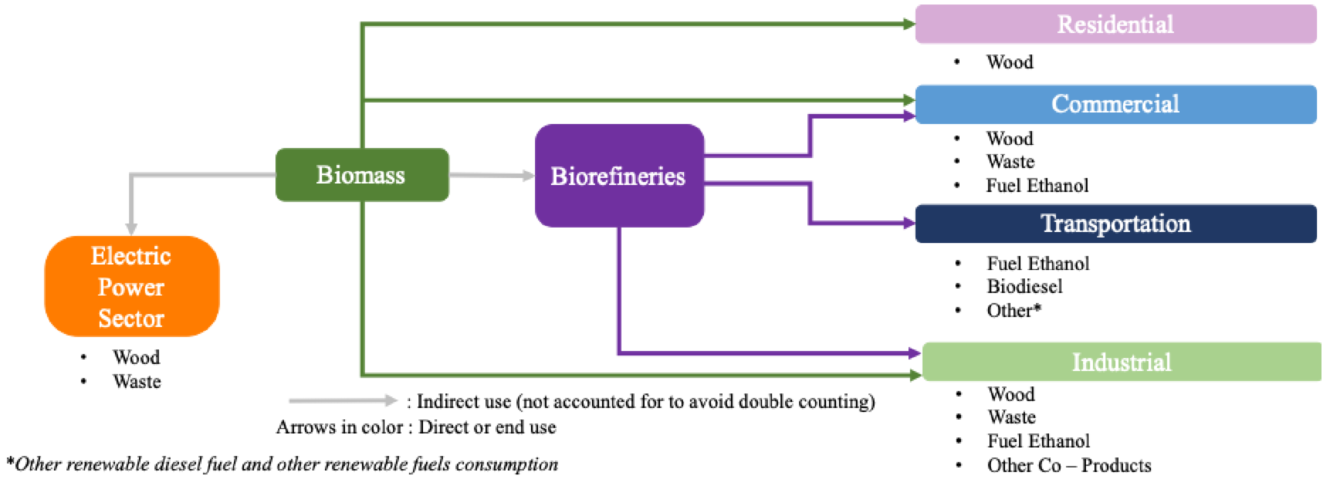

Supplementary Figure 2: Example of applying rolling horizon methodology up to 4 years for September 2020

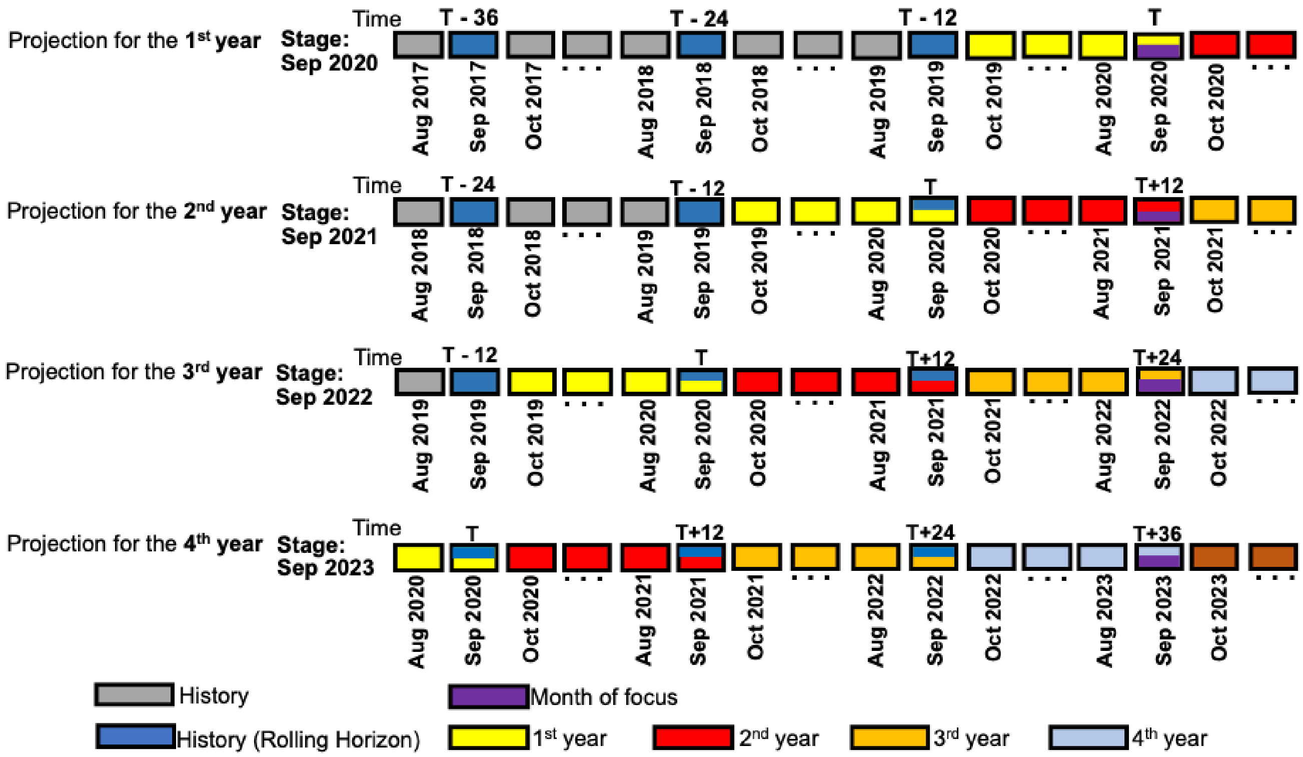

Supplementary Figure 3: Seasonal volatility of natural gas consumption

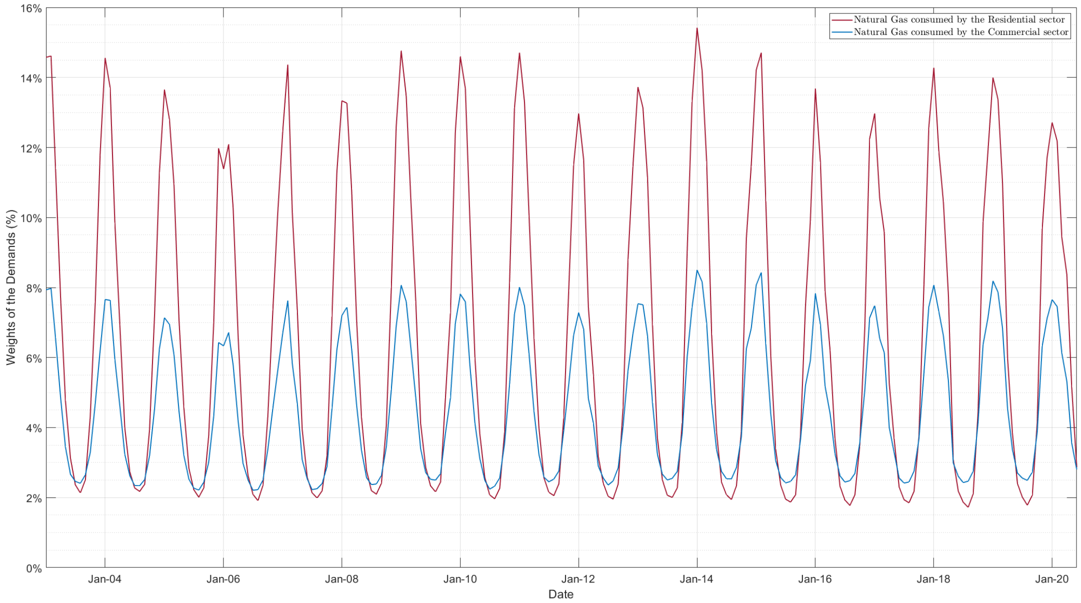

Energy demand is highly seasonal, so each month needs to be trained separately. The weights of the demands of natural gas consumed by the residential (red line) and commercial (blue line) sectors are shown over a period from 2003 to 2020. The higher weights for both sectors occur during the winter months, while the lower weights occur during the summer months. Source data are provided as a Source Data file.

27 **Supplementary Figure 4: Impact on EPIC from an increase in the federal tax on crude oil (January 2003 to June 2020)**

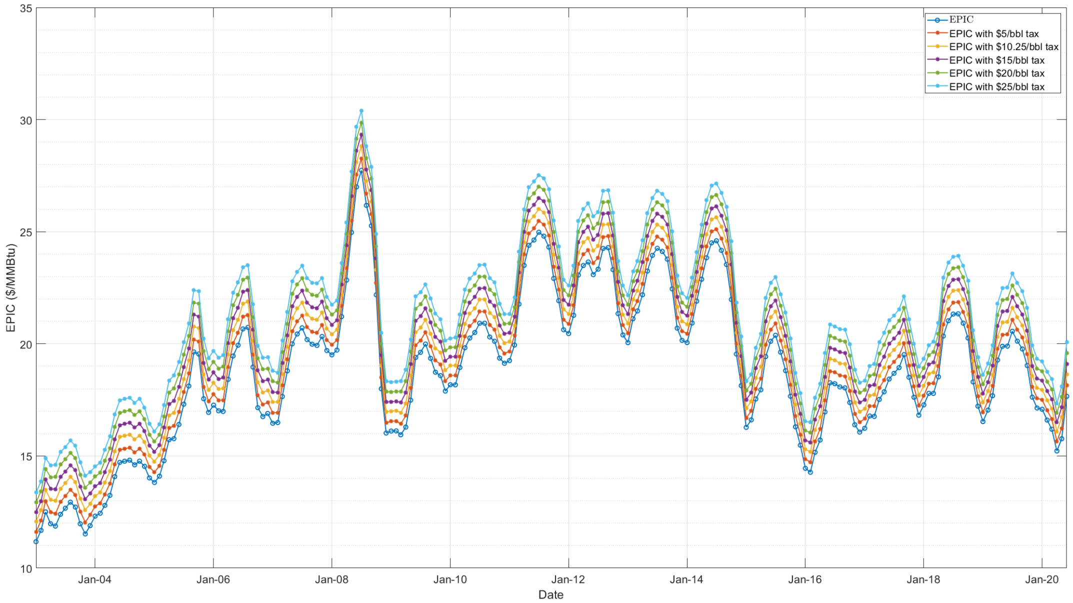

This figure illustrates the recalculated EPIC parametrically for different values of crude oil tax along with the reference value of EPIC without tax for easy comparison, over a period from January 2003 to June 2020. As expected, the higher the crude oil taxes, the greater the increase in EPIC values. Source data are provided as a Source Data file.

Supplementary Figure 5: Nuclear power at different target weights & tax credits (2020-2024)

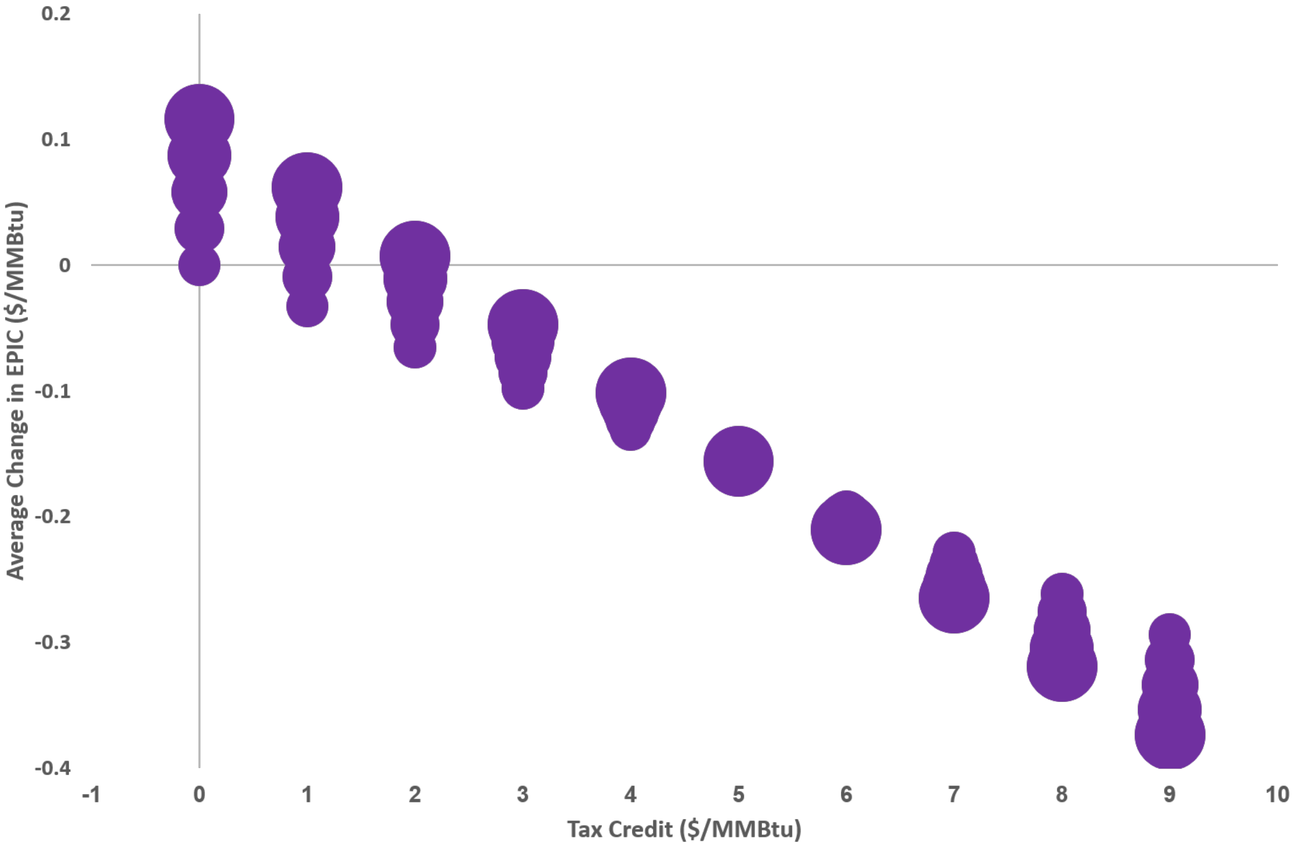

The grid of results for nuclear energy at different target weights and tax credits from 2020 to 2024 is shown in this figure. Due to the relatively higher levelized cost of nuclear energy, EPIC tends to increase at low tax credit levels, whereas EPIC decreases substantially (about $0.4/MMBtu) at the highest target weight (30%) and tax credit. At maximum weight (30%) and without tax credit, EPIC increases by 0.657% with no budget required, whereas at maximum weight (30%) and maximum tax credit ($9/MMBtu), EPIC decreases by 2.107% requiring around $38.0 billion annually from the government's budget. Source data are provided as a Source Data file.

Supplementary Figure 6: Hydroelectric power at different target weights & tax credits (2020-2024)

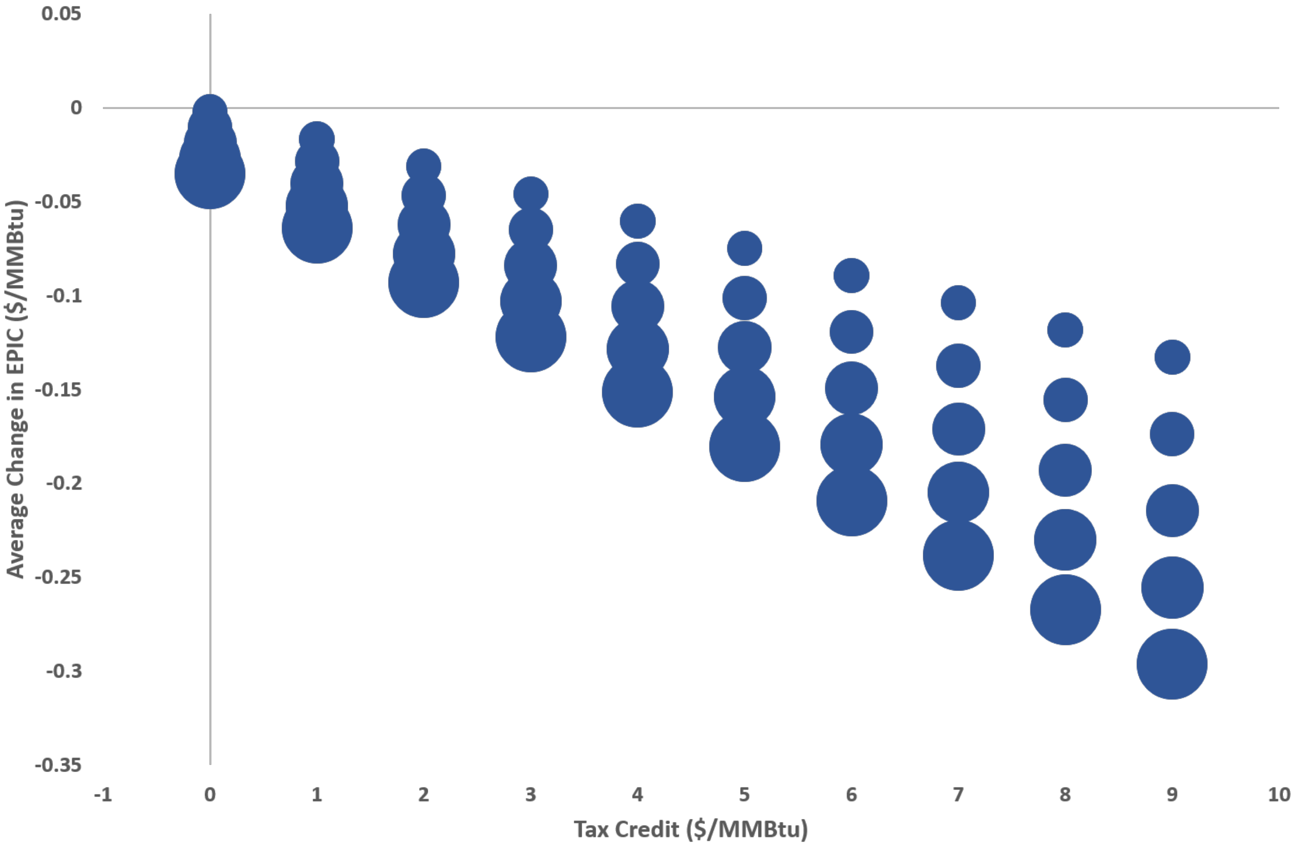

The grid of results for hydroelectric power at different target weights and tax credits from 2020 to 2024 is shown in this figure. Due to the low levelized cost of hydroelectric power, EPIC tends to decrease even without tax credit levels. At maximum weight (16%) and without tax credit, EPIC decreases by 0.198% with no budget required, whereas at maximum weight (16%) and maximum tax credit ($9/MMBtu), EPIC decreases by 1.672% requiring more than $20.0 billion annually from the government's budget. Source data are provided as a Source Data file.

Supplementary Figure 7: Biomass power at different target weights & tax credits (2020-2024)

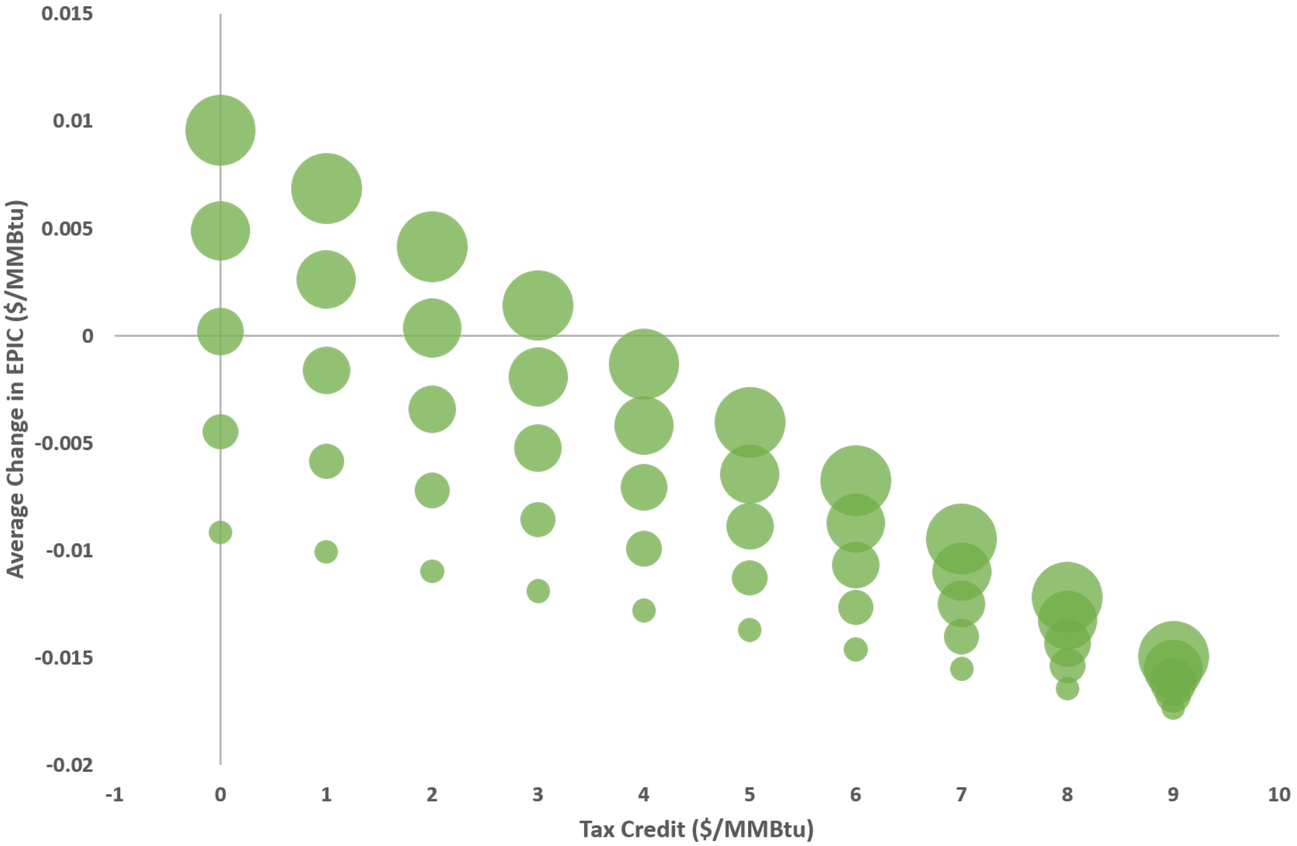

The grid of results for biomass energy at different target weights and tax credits from 2020 to 2024 is shown in this figure. The contribution of biomass into the electric power sector is rather low, even at the maximum weight target (1.5%). Due to the relatively higher levelized cost of biomass energy, EPIC decreases at higher tax credit. At maximum weight (1.5%) and without tax credit, EPIC increases by 0.054% with no budget required, whereas at maximum weight (1.5%) and maximum tax credit ($9/MMBtu), EPIC decreases by 0.084% requiring around $1.9 billion annually from the government's budget. Source data are provided as a Source Data file.

Supplementary Figure 8: Geothermal power at different target weights & tax credits (2020-2024)

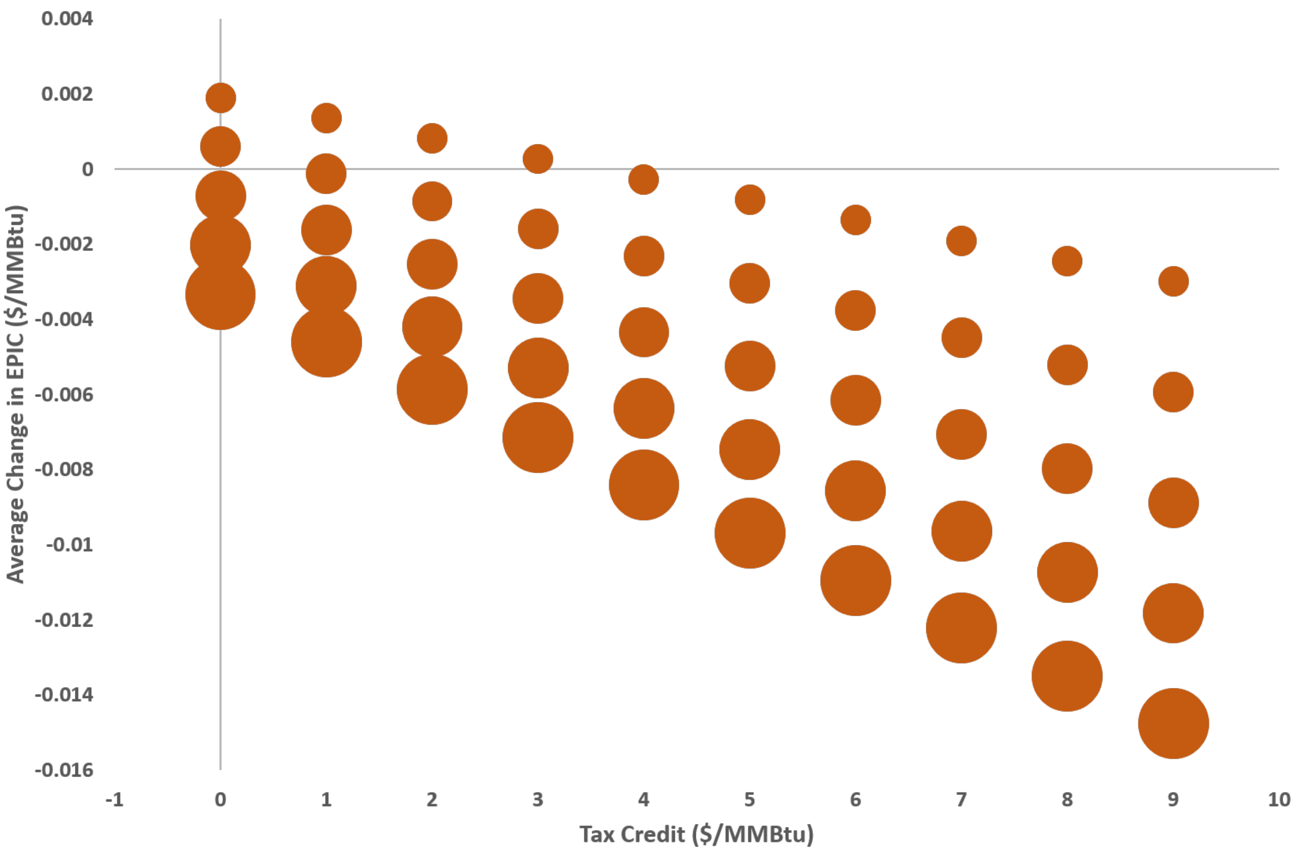

The grid of results for geothermal energy at different target weights and tax credits from 2020 to 2024 is shown in this figure. The contribution of geothermal into the electric power sector is limited, even at the maximum weight target (0.7%). Despite the fact that the levelized cost of geothermal energy is quite low, it has minimal effects on EPIC due to its limited availability as a source of energy in the power sector. At maximum weight (0.7%) and without tax credit, EPIC decreases by 0.019% with no budget required, whereas at maximum weight (0.7%) and maximum tax credit ($9/MMBtu), EPIC decreases by 0.083% requiring $887 million annually from the government's budget. Source data are provided as a Source Data file.

Supplementary Figure 9: Solar power at different target weights & tax credits (2020-2024)

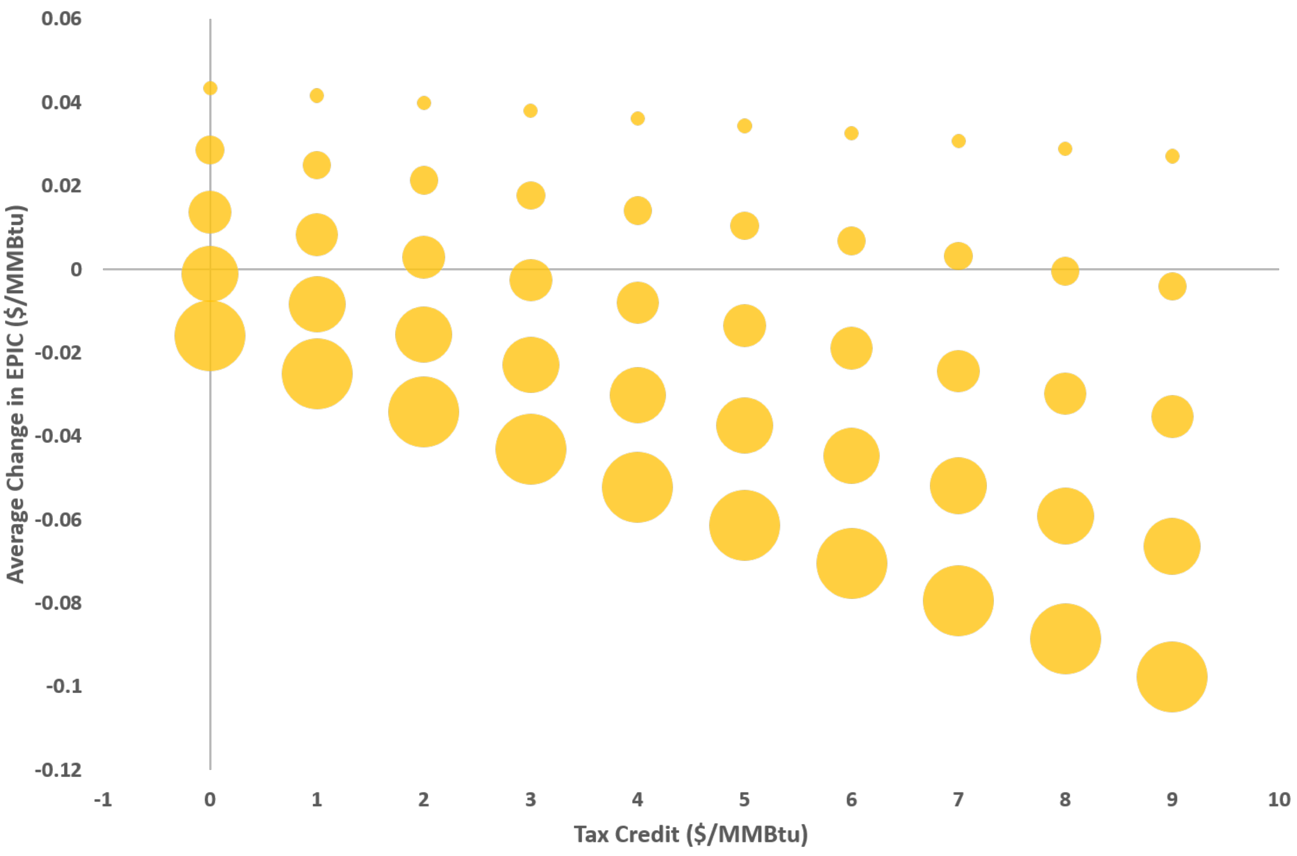

The grid of results for solar power at different target weights and tax credits from 2020 to 2024 is shown in this figure. The levelized cost of solar energy has decreased considerably over the years, resulting in decreasing EPIC even at low tax credit values. At maximum weight (5%) and without tax credit, EPIC decreases by 0.09% with no budget required, whereas at maximum weight (5%) and maximum tax credit ($9/MMBtu), EPIC decreases by 0.551% requiring more than $6.3 billion annually from the government's budget. Source data are provided as a Source Data file.

Supplementary Tables

Supplementary Table 1: Weights of various energy indices

**S&P 500 Energy Index [1]**	
Oil Gas & Consumable Fuel	91.00%
Energy Equipment & Services	9.00%

**S&P GSCI Energy Index [2]**	
WTI Crude Oil	41.01%
Brent Crude Oil	29.83%
RBOB Gasoline	7.34%
Heating Oil	6.92%
Gasoil	9.64%
Natural Gas	5.25%

**MSCI US IMI Energy 25/50 Index [3]**	
Integrated Oil & Gas	45.83%
Oil & Gas Exploration & Production	21.71%
Oil & Gas Refining & Marketing	12.31%
Oil & Gas Storage & Transportation	11.42%
Oil & Gas Equipment & Services	8.08%
Oil & Gas Drilling	0.52%
Coal & Consumable Fuels	0.14%

Supplementary Table 2: Energy products and sectors that are consumed in

Product No.	Product Name and Sector that is consumed in
1	Distillate fuel oil consumed by the residential sector
2	Kerosene consumed by the residential sector
3	Hydrocarbon gas liquids (Propane) consumed by the residential sector
4	Distillate fuel oil consumed by the commercial sector
5	Kerosene consumed by the commercial sector
6	Hydrocarbon gas liquids (Propane) consumed by the commercial sector
7	Motor gasoline consumed by the commercial sector
8	Petroleum coke consumed by the commercial sector
9	Residual fuel oil consumed by the commercial sector
10	Asphalt and Road oil consumed by the industrial sector
11	Distillate fuel oil consumed by the industrial sector
12	Kerosene consumed by the industrial sector
13	Hydrocarbon gas liquids (Propane/Propylene) consumed by the industrial sector
14	Lubricants consumed by the industrial sector
15	Motor gasoline consumed by the industrial sector
16	Petroleum coke consumed by the industrial sector
17	Residual fuel oil consumed by the industrial sector
18	Other petroleum products consumed by the industrial sector
19	Aviation gasoline consumed by the transportation sector
20	Distillate fuel oil consumed by the transportation sector
21	Jet fuel consumed by the transportation sector
22	Liquefied petroleum gases consumed by the transportation sector
23	Lubricants consumed by the transportation sector
24	Motor gasoline consumed by the transportation sector
25	Residual fuel oil consumed by the transportation sector
26	Geothermal energy consumed by the residential sector
27	Solar energy consumed by the residential sector
28	Biomass (wood) energy consumed by the residential sector
29	Hydroelectric power consumed by the commercial sector
30	Geothermal energy consumed by the commercial sector
31	Solar energy consumed by the commercial sector
32	Wind energy consumed by the commercial sector
33	Biomass (wood) energy consumed by the commercial sector
34	Biomass (waste) energy consumed by the commercial sector
35	Biomass (fuel ethanol)8 energy consumed by the commercial sector
36	Hydroelectric power consumed by the industrial sector
37	Geothermal energy consumed by the industrial sector
38	Solar energy consumed by the industrial sector
39	Wind energy consumed by the industrial sector
40	Biomass (wood) energy consumed by the industrial sector
41	Biomass (waste) energy consumed by the industrial sector
42	Biomass (fuel ethanol) energy consumed by the industrial sector
43	Biomass (losses and co-products) energy consumed by the industrial sector
44	Biomass (fuel ethanol) consumed by the transportation sector
45	Biomass (biodiesel) consumed by the transportation sector
46	Natural gas consumed by the residential sector
47	Natural gas consumed by the commercial sector
48	Natural gas consumed by the industrial sector
49	Natural gas consumed by the transportation sector
50	Electricity consumed by the residential sector
51	Electricity consumed by the commercial sector
52	Electricity consumed by the industrial sector
53	Electricity consumed by the transportation sector
54	Coal consumed by the residential sector
55	Coal consumed by the commercial sector
56	Coal consumed by the industrial sector

Supplementary Table 3: Weights of the demand of energy products in 2020

Product No.	January 2020	February 2020	March 2020	April 2020	May 2020	June 2020
1	0.83%	0.73%	0.74%	0.82%	0.91%	0.55%
2	0.05%	0.06%	0.02%	0.01%	0.00%	0.00%
3	1.05%	1.00%	0.88%	0.88%	0.55%	0.33%
4	0.53%	0.47%	0.47%	0.52%	0.58%	0.35%
5	0.01%	0.01%	0.00%	0.00%	0.00%	0.00%
6	0.32%	0.31%	0.29%	0.30%	0.23%	0.17%
7	0.44%	0.45%	0.45%	0.40%	0.50%	0.54%
8	0.00%	0.00%	0.00%	0.00%	0.00%	0.00%
9	0.00%	0.00%	0.00%	0.00%	0.00%	0.00%
10	0.59%	0.59%	0.73%	1.24%	1.57%	2.07%
11	1.86%	1.92%	1.79%	0.93%	0.75%	0.68%
12	0.01%	0.01%	0.00%	0.00%	0.00%	0.00%
13	1.07%	1.36%	1.28%	1.33%	1.45%	1.03%
14	0.16%	0.14%	0.10%	0.15%	0.15%	0.18%
15	0.32%	0.33%	0.33%	0.30%	0.37%	0.40%
16	0.59%	0.67%	0.71%	0.60%	0.72%	0.66%
17	0.04%	0.03%	0.02%	0.03%	0.02%	0.05%
18	3.37%	3.55%	4.00%	4.15%	4.41%	3.73%
19	0.03%	0.02%	0.02%	0.02%	0.04%	0.03%
20	7.42%	7.57%	9.08%	10.61%	10.88%	10.60%
21	4.41%	4.30%	4.24%	2.51%	2.19%	2.72%
22	0.01%	0.01%	0.01%	0.01%	0.01%	0.01%
23	0.18%	0.16%	0.11%	0.17%	0.17%	0.20%
24	19.83%	20.32%	20.39%	18.27%	22.65%	24.59%
25	0.63%	0.35%	0.28%	0.39%	0.23%	0.74%
26	0.05%	0.05%	0.06%	0.07%	0.07%	0.07%
27	0.24%	0.29%	0.42%	0.58%	0.63%	0.62%
28	0.63%	0.63%	0.73%	0.87%	0.88%	0.83%
29	0.00%	0.00%	0.00%	0.00%	0.00%	0.00%
30	0.03%	0.03%	0.03%	0.03%	0.03%	0.03%
31	0.10%	0.13%	0.18%	0.24%	0.26%	0.26%
32	0.00%	0.00%	0.00%	0.00%	0.00%	0.00%
33	0.11%	0.11%	0.12%	0.14%	0.15%	0.14%
34	0.05%	0.05%	0.06%	0.06%	0.06%	0.06%
35	0.03%	0.03%	0.03%	0.03%	0.04%	0.04%
36	0.01%	0.01%	0.02%	0.02%	0.02%	0.02%
37	0.01%	0.01%	0.01%	0.01%	0.01%	0.01%
38	0.03%	0.03%	0.05%	0.06%	0.07%	0.07%
39	0.00%	0.00%	0.00%	0.00%	0.00%	0.01%
40	1.78%	1.77%	1.98%	2.40%	2.46%	2.17%
41	0.22%	0.22%	0.25%	0.30%	0.29%	0.24%
42	0.02%	0.02%	0.02%	0.02%	0.03%	0.03%
43	1.05%	1.03%	1.08%	0.76%	0.93%	1.11%
44	1.42%	1.38%	1.36%	1.14%	1.63%	1.80%
45	0.25%	0.31%	0.31%	0.40%	0.40%	0.39%
46	12.71%	12.19%	9.43%	8.36%	5.15%	2.87%
47	7.65%	7.45%	6.12%	5.32%	3.56%	2.80%
48	12.23%	12.13%	12.94%	14.25%	13.45%	12.73%
49	0.08%	0.08%	0.09%	0.11%	0.11%	0.10%
50	6.33%	6.14%	6.16%	7.11%	7.51%	9.10%
51	5.51%	5.59%	6.10%	6.61%	6.65%	7.54%
52	3.96%	4.14%	4.59%	5.07%	5.08%	5.22%
53	0.04%	0.03%	0.04%	0.03%	0.03%	0.03%
54	0.00%	0.00%	0.00%	0.00%	0.00%	0.00%
55	0.03%	0.04%	0.03%	0.02%	0.02%	0.02%
56	1.64%	1.74%	1.85%	2.31%	2.11%	2.05%

Supplementary Table 4: Demands & prices of energy products

Product No.	Demand Data	Price Data
1	EIA MER[4]: Table 3.8a	EIA Petroleum and Other Liquids
2	EIA MER[4]: Table 3.8a	EIA MER[4]: Table 9.7
3	EIA MER[4]: Table 3.8a	EIA Petroleum and Other Liquids
4	EIA MER[4]: Table 3.8a	EIA MER[4]: Table 9.7
5	EIA MER[4]: Table 3.8a	EIA MER[4]: Table 9.7
6	EIA MER[4]: Table 3.8a	EIA MER[4]: Table 9.7
7	EIA MER[4]: Table 3.8a	EIA MER[4]: Table 9.7
8	EIA MER[4]: Table 3.8a	EIA EPM[5]: Table 4.1 & 4.2
9	EIA MER[4]: Table 3.8a	EIA MER[4]: Table 9.5
10	EIA MER[4]: Table 3.8b	EIA SEDS: Table F2[6] & BLS Database[7]
11	EIA MER[4]: Table 3.8b	EIA MER[4]: Table 9.7
12	EIA MER[4]: Table 3.8b	EIA MER[4]: Table 9.7
13	EIA MER[4]: Table 3.8b	EIA MER[4]: Table 9.7
14	EIA MER[4]: Table 3.8b	EIA SEDS: Table F10[6] & BLS Database[7]
15	EIA MER[4]: Table 3.8b	EIA MER[4]: Table 9.7
16	EIA MER[4]: Table 3.8a	EIA EPM[5]: Table 4.1 & 4.2
17	EIA MER[4]: Table 3.8b	EIA MER[4]: Table 9.5
18	EIA MER[4]: Table 3.8b	EIA SEDS: Table F15[6] & BLS Database[7]
19	EIA MER[4]: Table 3.8c	EIA MER[4]: Table 9.6 & 9.7
20	EIA MER[4]: Table 3.8c	EIA MER[4]: Table 9.7
21	EIA MER[4]: Table 3.8c	EIA MER[4]: Table 9.7
22	EIA MER[4]: Table 3.8c	EIA MER[4]: Table 9.7
23	EIA MER[4]: Table 3.8c	EIA SEDS: Table F10[6] & BLS Database[7]
24	EIA MER[4]: Table 3.8c	EIA MER[4]: Table 9.4
25	EIA MER[4]: Table 3.8c	EIA MER[4]: Table 9.5
26	EIA MER[4]: Table 10.2a	Lazard LCEA[8]
27	EIA MER[4]: Table 10.2a	Lazard LCEA[8]
28	EIA MER[4]: Table 10.2a	Lazard LCEA[8] and EIA AEO[9]
29	EIA MER[4]: Table 10.2a	EIA AEO[9]
30	EIA MER[4]: Table 10.2a	Lazard LCEA[8]
31	EIA MER[4]: Table 10.2a	Lazard LCEA[8]
32	EIA MER[4]: Table 10.2a	Lazard LCEA[8]
33	EIA MER[4]: Table 10.2a	Lazard LCEA[8] & EIA AEO[9]
34	EIA MER[4]: Table 10.2a	Lazard LCEA[8] & EIA AEO[9]
35	EIA MER[4]: Table 10.2a	EIA MER[4]: Table 9.7 & DOE AFPR[10]
36	EIA MER[4]: Table 10.2b	EIA AEO[9]
37	EIA MER[4]: Table 10.2b	Lazard LCEA[8]
38	EIA MER[4]: Table 10.2b	Lazard LCEA[8]
39	EIA MER[4]: Table 10.2b	Lazard LCEA[8]
40	EIA MER[4]: Table 10.2b	Lazard LCEA[8] & EIA AEO[9]
41	EIA MER[4]: Table 10.2b	Lazard LCEA[8] & EIA AEO[9]
42	EIA MER[4]: Table 10.2a	EIA MER[4]: Table 9.7 & DOE AFPR[10]
43	EIA MER[4]: Table 10.2b	Lazard LCEA[8] & EIA AEO[9]
44	EIA MER[4]: Table 10.2a	EIA MER[4]: Table 9.7 & DOE AFPR[10]
45	EIA MER[4]: Table 10.2a	EIA MER[4]: Table 9.7 & DOE AFPR[10]
46	EIA MER[4]: Table 4.3	EIA MER[4]: Table 9.10
47	EIA MER[4]: Table 4.3	EIA MER[4]: Table 9.10
48	EIA MER[4]: Table 4.3	EIA MER[4]: Table 9.10
49	EIA MER[4]: Table 4.3	EIA SEDS: Table F19[6] & Thomson Reuters Database[11]
50	EIA EPM[5]: Table 5.1	EIA EPM[5]: Table 5.3
51	EIA EPM[5]: Table 5.1	EIA EPM[5]: Table 5.3
52	EIA EPM[5]: Table 5.1	EIA EPM[5]: Table 5.3
53	EIA EPM[5]: Table 5.1	EIA EPM[5]: Table 5.3
54	EIA MER[4]: Table 6.2	EIA SEDS: Table F24[6] & BLS Database[7]
55	EIA MER[4]: Table 6.2	EIA SEDS: Table F24[6] & BLS Database[7]
56	EIA MER[4]: Table 6.2	EIA SEDS: Table F24[6] & BLS Database[7]

Supplementary Table 5: Real weights of natural gas consumption by the residential and commercial sectors[4]

Date	Real weight of natural gas consumed by the residential sector	Real weight of natural gas consumed by the commercial Sector
2015 April	6.022%	4.379%
2015 May	3.383%	3.058%
2015 June	2.357%	2.573%
2015 July	1.957%	2.419%
2015 August	1.871%	2.461%
2015 September	2.082%	2.651%
2015 October	3.766%	3.643%
2015 November	7.471%	5.204%
2015 December	9.904%	5.908%
2016 January	13.680%	7.830%
2016 February	11.583%	6.941%
2016 March	7.896%	5.179%
2016 April	6.190%	4.397%
2016 May	3.684%	3.255%
2016 June	2.323%	2.621%
2016 July	1.931%	2.443%
2016 August	1.776%	2.483%
2016 September	2.079%	2.690%
2016 October	3.490%	3.565%
2016 November	6.861%	5.070%
2016 December	12.247%	7.135%
2017 January	12.949%	7.472%
2017 February	10.522%	6.528%
2017 March	9.570%	6.134%
2017 April	5.256%	3.994%
2017 May	3.669%	3.283%
2017 June	2.298%	2.558%
2017 July	1.938%	2.411%
2017 August	1.849%	2.444%
2017 September	2.176%	2.752%
2017 October	3.740%	3.660%
2017 November	8.063%	5.559%
2017 December	12.551%	7.436%
2018 January	14.266%	8.060%
2018 February	11.870%	7.304%
2018 March	10.427%	6.613%
2018 April	7.767%	5.321%
2018 May	3.080%	2.985%
2018 June	2.178%	2.602%
2018 July	1.867%	2.425%
2018 August	1.719%	2.465%
2018 September	2.104%	2.747%
2018 October	4.450%	4.141%
2018 November	9.871%	6.370%
2018 December	11.886%	7.132%
2019 January	14.033%	8.210%
2019 February	13.408%	7.889%
2019 March	10.990%	6.833%
2019 April	6.027%	4.571%
2019 May	3.855%	3.384%
2019 June	2.407%	2.707%
2019 July	2.015%	2.557%
2019 August	1.777%	2.486%
2019 September	2.066%	2.720%

Supplementary Table 6: Weights and levelized cost of energy feedstocks for the electric power sector

The weights of the different energy feedstocks are taken from the EIA Monthly Energy Review [4] for the period from January 2003 to June 2020. The levelized cost of the energy feedstocks is taken from the Lazard's Levelized Cost of Energy Analysis report for the period from 2008 to 2013 [12–17] and the EIA Annual Energy Outlook for the period 2014 to 2020 [18–24] apart from the figures for the petroleum liquids which are also taken from the Lazard's Levelized Cost of Energy Analysis reports. The data from 2008 are used for the period from 2003 to 2007.

Feedstock	Average weight (%)	Minimum weight (%)	Maximum weight (%)	LCOE (2020 $/MMBtu)
Coal	40.95	15.29	54.97	22.40
Natural Gas	25.21	11.72	43.12	15.34
Petroleum Liquids	1.26	0.42	4.45	67.78
Nuclear	20.45	17.27	22.93	21.95
Hydroelectric	7.03	4.28	10.68	15.47
Wind	3.51	0.19	11.24	11.71
Biomass	0.71	0.54	0.95	27.79
Solar	0.48	0.00083	3.32	9.71
Geothermal	0.40	0.31	0.50	10.38

Supplementary Notes

Supplementary Note 1: U.S end-use sectors

The definitions of each of the four end-use sectors and the electric power intermediate energy - consuming sector are provided verbatim by EIA [4, 25] as follows:

- **Residential Sector:** An energy – consuming sector that consists of living quarters for private households. Common uses of energy associated with this sector include space heating, water heating, air conditioning, lighting, refrigeration, cooking, and running a variety of other appliances. The residential sector excludes institutional living quarters.
- **Commercial Sector:** An energy – consuming sector that consists of service-providing facilities and equipment of businesses; Federal, State, and local governments; and other private and public organisations, such as religious, social, or fraternal groups. The commercial sector includes institutional living quarters. It also includes sewage treatment facilities. Common uses of energy associated with this sector include space heating, water heating, air conditioning, lighting, refrigeration, cooking, and running a wide variety of other equipment. *Note:* This sector includes generators that produce electricity and/or useful thermal output primarily to support the activities of the above-mentioned commercial establishments.
- **Industrial Sector:** An energy – consuming sector that consists of all facilities and equipment used for producing, processing, or assembling goods. The industrial sector encompasses the following types of activity manufacturing (NAICS[1]codes 31-33); agriculture, forestry, fishing and hunting (NAICS code 11); mining, including oil and gas extraction (NAICS code 21); and construction (NAICS code 23). Overall energy use in this sector is largely for process heat and cooling and powering machinery, with lesser amounts used for facility heating, air conditioning, and lighting. Fossil fuels are also used as raw material inputs to manufactured products. *Note:* This sector includes generators that produce electricity and/or useful thermal output primarily to support the above mentioned industrial activities..
- **Transportation Sector:** An energy – consuming sector that consists of all vehicles whose primary purpose is transporting people and/or goods from one physical location to another. Included are automobiles; trucks; buses; motorcycles; trains, subways, and other rail vehicles; aircraft; and ships, barges, and other waterborne vehicles. Vehicles whose primary purpose is not transportation (e.g., construction cranes and bulldozers, farming vehicles, and warehouse tractors and forklifts) are classified in the sector of their primary use.
- **Electric power sector:** An energy – consuming sector that consists of electricity only and combined heat and power (CHP) plants whose primary business is to sell electricity, or electricity and heat, to the public –i.e., North American Industry Classification System 22 plants.

Supplementary Note 2: Extra Details for the Demands of the Energy Products

- Demand data for Products 1 to 45 are provided in Trillion BTUs for each month.
- Natural Gas Consumption (Products 46-49) are provided in Billion Cubic Feet for each month. A conversion factor of 1,036 BTU per cubic foot is used to convert into units of energy. (Table A4)[4]
- Electricity Consumption (Products 50-53) are provided in Thousand Megawatt hours for each month. A conversion factor of 3,412 BTU per kWh is used to convert into units of energy. (Table A6)[4]
- Coal Consumption (Products 54-56) are provided in Thousand Short Tons for each month. For the residential and commercial coal consumption (Products 54-55), a conversion factor of 19.268 MMBTU per short ton is used to convert into units of energy, while for the industrial coal consumption (Product 56) a conversion factor of 28.608 MMBTU per short ton is used. (Table A5)[4]

[1]The North American Industry Classification System (NAICS) is the standard used by Federal statistical agencies in classifying business establishments for the purpose of collecting, analysing, and publishing statistical data related to the U.S. business economy. It was developed jointly by the U.S. Economic Classification Policy Committee (ECPC), Statistics Canada , and Mexico's Instituto Nacional de Estadistica y Geografia , to allow for a high level of comparability in business statistics among the North American countries. (https://www.census.gov/eos/www/naics/).

Supplementary Note 3: Extra Details for the Prices of the Energy Products

1. Distillate fuel oil consumed by the residential sector
    (a) The U.S. No. 2 Heating Oil Residential prices (Dollars per Gallon) are used. Prices are given for 6 months of the year (October – March). For the rest of the months, the No. 2 Heating Oil New York Harbor Spot Prices ($/gal) are used to estimate the distillate fuel oil prices using linear regression.
    (b) A conversion factor of 138,490 BTU/gal is used.
    (c) Federal tax and average state tax are added on top of the above-mentioned prices. (https://www.eia.gov/petroleum/marketing/monthly/xls/fueltaxes.xls)
2. Kerosene consumed by the residential sector
    (a) The prices of U.S. Kerosene Retail Sales by Refiners ($/gal) are used. If a price is not available for a month, then the prices of the U.S. Kerosene-Type Jet Fuel Retail Sales by Refiners are used to estimate the price of price using linear regression.
    (b) A conversion factor of 0.135 MMBTU/gal is used.
    (c) Federal tax and average state tax are added on top of the above-mentioned prices. (https://www.eia.gov/petroleum/marketing/monthly/xls/fueltaxes.xls)
3. Hydrocarbon gas liquids (Propane) consumed by the residential sector
    (a) The U.S. Propane Residential prices (Dollars per Gallon) are used. Prices are given for 6 months of the year (October – March). For the rest of the months, the Mont Belvieu, TX Propane Spot Price FOB ($/gal) are used to estimate the residential hydrocarbon gas liquid prices using linear regression.
    (b) A conversion factor of 0.09133 MMBtu/gal is used.
    (c) Federal tax is added on top of the above-mentioned prices. (https://afdc.energy.gov/fuels/laws/LPG?state=us)
4. Distillate fuel oil consumed by the commercial sector
    (a) The U.S. No. 2 Fuel Oil Retail Sales by Refiners (Dollars per Gallon) are used.
    (b) A conversion factor of 0.13849 MMBtu/gal is used.
    (c) Federal tax and average state tax are added on top of the above-mentioned prices. (https://www.eia.gov/petroleum/marketing/monthly/xls/fueltaxes.xls)

10. Asphalt and Road oil consumed by the industrial sector
    (a) The Asphalt and road oil average prices, for all end-use sectors in the United States (Dollars per MMBtu) are used from State Energy Data System (SEDS). [6]. Since the data are presented annually, their monthly values are estimated through a linear regression with the data of the Producer Price Index (PPI) of Asphalt (PPI by Industry: Petroleum Refineries: Asphalt).
    (b) No tax is added as per the SEDS.

19. Aviation gasoline consumed by the transportation sector
    (a) The U.S. Aviation Gasoline Retail Sales by Refiners (Dollars per Gallon) are used. In case there are no available data for a month, a linear regression of the retail sales and the U.S. Aviation Gasoline Wholesale/Resale Price by Refiners (Dollars per Gallon) is used to determine the retail price of that month.
    (b) A conversion factor of 0.12019 MMBtu/gal is used.
    (c) No tax is added as per the SEDS.

26. Geothermal energy consumed by the residential sector
    (a) The levelized cost of energy ($/MWh) from Lazard's Levelized Cost of Energy Analysis report is used. The average value between the low and the high values of geothermal that are provided in the report is used.

(b) A conversion factor of 0.29308 MWh/MMBtu is used.

(c) A constant value is taken for all months of the year.

47. Natural gas consumed by the commercial sector

    (a) The U.S. Price of Natural Gas Sold to Commercial Consumers (Dollars per Thousand Cubic Feet) is used.

    (b) A conversion factor of 1.036 Million Btu/Thousand cubic Feet is used.

    (c) Taxes are included in the price.

52. Electricity consumed by the industrial sector

    (a) The average retail price of electricity in the industrial sector (cents per kWh) is used.

    (b) A conversion factor of 0.003412 Million Btu/kWh is used.

    (c) Taxes are included in the price.

55. Coal consumed by the commercial sector

    (a) The Coal price in the commercial sector (Dollar per MMBtu) is used. Since the data are presented annually, their monthly values are estimated through a linear regression with the data of the Producer Price Index (PPI) by Commodity for Fuels and Related Products and Power: Coal.

    (b) Taxes are included in the price.

References

1. Indices, D. J. S. *S&P U.S. Indices Methodology* tech. rep. (May 2020).
2. Indices, D. J. S. *S&P GSCI Methodology* tech. rep. (May 2020).
3. MSCI. *MSCI U.S. IMI Energy 25/50 Index* tech. rep. (May 2020).
4. U.S. Energy Information Administration. *Monthly Energy Review* tech. rep. (Washington, DC, USA, September 2020).
5. U.S. Energy Information Administration. *Electric Power Monthly with Data for July 2020* tech. rep. (Washington, DC, USA, September 2020).
6. U.S. Energy Information Administration. *State Energy Data System 2017* tech. rep. (Washington, DC, USA, 2017).
7. U.S. Department of Labor. *Bureau of Labor Statistics, Producer Price Index, November 2019* tech. rep. (Washington, DC, USA, November 2019).
8. Lazard. *Lazard's Levelized Cost of Energy Analysis, Version 13.0* tech. rep. (New York, USA, November 2019).
9. U.S. Energy Information Administration. *Annual Energy Outlook 2020 with projections to 2050* tech. rep. (Washington, DC, USA, 2020).
10. U.S. Department of Energy. *Clean Cities - Alternative Fuel Price Report* tech. rep. (Washington, DC, USA, April 2020).
11. Thomson Reuters Database. *Henry Hub Natural Gas Spot Price* 2020.
12. Lazard. *Lazard's Levelized Cost of Energy Analysis, Version 2.0* tech. rep. (New York, USA, June 2008).
13. Lazard. *Lazard's Levelized Cost of Energy Analysis, Version 3.0* tech. rep. (New York, USA, February 2009).
14. Lazard. *Lazard's Levelized Cost of Energy Analysis, Version 4.0* tech. rep. (New York, USA, June 2010).
15. Lazard. *Lazard's Levelized Cost of Energy Analysis, Version 5.0* tech. rep. (New York, USA, June 2011).
16. Lazard. *Lazard's Levelized Cost of Energy Analysis, Version 6.0* tech. rep. (New York, USA, June 2012).
17. Lazard. *Lazard's Levelized Cost of Energy Analysis, Version 7.0* tech. rep. (New York, USA, August 2013).
18. U.S. Energy Information Administration. *Levelized Cost and Levelized Avoided Cost of New Generation Resources in the Annual Energy Outlook 2014* tech. rep. (Washington, DC, USA, April 2014).
19. U.S. Energy Information Administration. *Levelized Cost and Levelized Avoided Cost of New Generation Resources in the Annual Energy Outlook 2015* tech. rep. (Washington, DC, USA, June 2015).
20. U.S. Energy Information Administration. *Levelized Cost and Levelized Avoided Cost of New Generation Resources in the Annual Energy Outlook 2016* tech. rep. (Washington, DC, USA, August 2016).
21. U.S. Energy Information Administration. *Levelized Cost and Levelized Avoided Cost of New Generation Resources in the Annual Energy Outlook 2017* tech. rep. (Washington, DC, USA, April 2017).
22. U.S. Energy Information Administration. *Levelized Cost and Levelized Avoided Cost of New Generation Resources in the Annual Energy Outlook 2018* tech. rep. (Washington, DC, USA, March 2018).
23. U.S. Energy Information Administration. *Levelized Cost and Levelized Avoided Cost of New Generation Resources in the Annual Energy Outlook 2019* tech. rep. (Washington, DC, USA, February 2019).
24. U.S. Energy Information Administration. *Levelized Cost and Levelized Avoided Cost of New Generation Resources in the Annual Energy Outlook 2020* tech. rep. (Washington, DC, USA, February 2020).
25. U.S. Energy Information Administration. *Glossary, Energy Information Administration* https://www.eia.gov/tools/glossary/ (Accessed: 12.12.2019). 2019.
