# Supplementary material for: A framework to predict the price of energy for the end-users with applications to monetary and energy policies
Source: Nat Commun. 2021 Jan 4;12:18. doi: 10.1038/s41467-020-20203-2 (PMC7782726; doi:10.1038/s41467-020-20203-2)
Supplement: Supplementary file 3 — Reporting Summary [file 41467_2020_20203_MOESM3_ESM.pdf]

nature research

Corresponding author(s): Stefanos G. Baratsas, Alexander M. Niziolek, Onur Onel, Logan R. Matthews, Christodoulos A. Floudas, Detlef R. Hallermann, Sorin M. Sorescu, and Efstratios N. Pistikopoulos

Last updated by author(s): Oct 5, 2020

Reporting Summary

Nature Research wishes to improve the reproducibility of the work that we publish. This form provides structure for consistency and transparency in reporting. For further information on Nature Research policies, see our Editorial Policies and the Editorial Policy Checklist.

Statistics

For all statistical analyses, confirm that the following items are present in the figure legend, table legend, main text, or Methods section.

n/a	Confirmed	
☒	☐	The exact sample size (n) for each experimental group/condition, given as a discrete number and unit of measurement
☒	☐	A statement on whether measurements were taken from distinct samples or whether the same sample was measured repeatedly
☒	☐	The statistical test(s) used AND whether they are one- or two-sided<br>*Only common tests should be described solely by name; describe more complex techniques in the Methods section.*
☒	☐	A description of all covariates tested
☒	☐	A description of any assumptions or corrections, such as tests of normality and adjustment for multiple comparisons
☒	☐	A full description of the statistical parameters including central tendency (e.g. means) or other basic estimates (e.g. regression coefficient) AND variation (e.g. standard deviation) or associated estimates of uncertainty (e.g. confidence intervals)
☒	☐	For null hypothesis testing, the test statistic (e.g. F, t, r) with confidence intervals, effect sizes, degrees of freedom and P value noted<br>*Give P values as exact values whenever suitable.*
☒	☐	For Bayesian analysis, information on the choice of priors and Markov chain Monte Carlo settings
☒	☐	For hierarchical and complex designs, identification of the appropriate level for tests and full reporting of outcomes
☒	☐	Estimates of effect sizes (e.g. Cohen's d, Pearson's r), indicating how they were calculated

Our web collection on statistics for biologists contains articles on many of the points above.

Software and code

Policy information about availability of computer code

Data collection	All data are collected in excel files. Energy data from EIA's website were collected using the Microsoft Excel Data Add-In webtool. All data sources are presented in Supplementary Table 4
Data analysis	The optimization code in GAMS that supports the analysis within this paper and other findings of this study are available from the corresponding author upon reasonable request.

For manuscripts utilizing custom algorithms or software that are central to the research but not yet described in published literature, software must be made available to editors and reviewers. We strongly encourage code deposition in a community repository (e.g. GitHub). See the Nature Research guidelines for submitting code & software for further information.

Data

Policy information about availability of data

All manuscripts must include a data availability statement. This statement should provide the following information, where applicable:

- Accession codes, unique identifiers, or web links for publicly available datasets
- A list of figures that have associated raw data
- A description of any restrictions on data availability

The source data underlying Figures 2, 4, 5 and Supplementary Figures 3 to 9 are provided as a Source Data file. Figures 1, 3 and Supplementary Figures 1 and 2 do not have associated data. All data used for this analysis are available from cited publicly available sources or from the corresponding author upon reasonable request.

Field-specific reporting

Please select the one below that is the best fit for your research. If you are not sure, read the appropriate sections before making your selection.

☐ Life sciences ☐ Behavioural & social sciences ☒ Ecological, evolutionary & environmental sciences

For a reference copy of the document with all sections, see nature.com/documents/nr-reporting-summary-flat.pdf

Ecological, evolutionary & environmental sciences study design

All studies must disclose on these points even when the disclosure is negative.

Study description	This study presents a novel framework, an index to calculate the average price of energy in the United States. The complex energy landscape is thoroughly analysed to accurately determine the two key factors of this framework: the total demand of the energy products directed to the end-use sectors, and the corresponding price of each product. The effectiveness of the framework is demonstrated by addressing two policy question of significant public interest.
Research sample	The monthly consumption (in energy units) along with the monthly price (in $ per energy unit) for each of the energy products is extracted from the data provided by the EIA and other sources and are shown in the Supplementary Table 4.
Sampling strategy	No sample size calculation was performed. Sizes were determined by the data availability from all various sources of all the involved parameters. Since the proposed index represents the the average price of energy in the US, a period of 15 years was set as the minimum in data collection. Therefore, data from January 2003 to June 2020 have been used for this study.
Data collection	Data are collected from cited publicly available sources, reports, and electronic databases. All data are collected in excel files. Energy data from EIA's website were collected using the Microsoft Excel Data Add-In webtool. Supplementary Notes 2 and 3 provide step by step details of the preliminary calculations for the preparation of the data to be used in the framework. Stefanos G. Baratsas, Alexander M. Niziolek, Onur Onel, and Logan R. Matthews have been working on the data collection.
Timing and spatial scale	The historical data refer to the period from January 2003 to June 2020, while the predicted data refer to the period from July 2020 up to June 2024. The frequency of data is monthly. The data of the demands and the prices refer to the energy products in the U.S. energy landscape and are captured through 56 different energy products.
Data exclusions	No data are excluded.
Reproducibility	No experiments took place.
Randomization	All available data over the specified period from 2003 to 2020 have been used for this study and analysis.
Blinding	All available data over the specified period from 2003 to 2020 have been used for this study and analysis.

Did the study involve field work? ☐ Yes ☒ No

Reporting for specific materials, systems and methods

We require information from authors about some types of materials, experimental systems and methods used in many studies. Here, indicate whether each material, system or method listed is relevant to your study. If you are not sure if a list item applies to your research, read the appropriate section before selecting a response.

Materials & experimental systems

n/a	Involved in the study
☒	☐ Antibodies
☒	☐ Eukaryotic cell lines
☒	☐ Palaeontology and archaeology
☒	☐ Animals and other organisms
☒	☐ Human research participants
☒	☐ Clinical data
☒	☐ Dual use research of concern

Methods

n/a	Involved in the study
☒	☐ ChIP-seq
☒	☐ Flow cytometry
☒	☐ MRI-based neuroimaging
